# Development and validation of a CT-based multi-omics nomogram for predicting hospital discharge outcomes following mechanical thrombectomy

**DOI:** 10.3389/fnins.2025.1643014

**Published:** 2025-08-07

**Authors:** Feifan Liu, Jiayi Hong, Yuhan Chen, Huan Liu, Yue Wang, Lijian Su, Sheng Hu, Jingjing Fu

**Affiliations:** ^1^Department of Neurology, The Fourth Affiliated Hospital of School of Medicine, and International School of Medicine, International Institutes of Medicine, Zhejiang University, Yiwu, China; ^2^Department of Radiology, The Fourth Affiliated Hospital of School of Medicine, and International School of Medicine, International Institutes of Medicine, Zhejiang University, Yiwu, China

**Keywords:** prognosis, nomogram, thrombectomy, acute ischemic stroke, multi-detector CT

## Abstract

**Objective:**

This study aimed to develop a multi-omics nomogram that combines clinical parameters, radiomics, and deep transfer learning (DTL) features of hyperattenuated imaging markers (HIM) from computed tomography scans immediately following mechanical thrombectomy (MT) to predict functional outcomes at discharge.

**Methods:**

This study enrolled 246 patients with HIM who underwent MT. Patients were randomly assigned to a training cohort (*n* = 197, 80%) and a validation cohort (*n* = 49, 20%), with an additional internal prospective test cohort (*n* = 57). A total of 1,834 radiomics features and 25,088 DTL features were extracted from HIM images. Feature selection was conducted using analysis of variance (ANOVA), Pearson’s correlation, principal component analysis (PCA), and least absolute shrinkage and selection operator (LASSO) regression. A support vector machine (SVM)-based nomogram integrating clinical, radiomics, and DTL features was developed to predict functional outcomes at discharge. Its performance was evaluated based on accuracy, sensitivity, specificity, receiver operating characteristic (ROC) curve and area under the curve (AUC) analysis, decision curve analysis (DCA), and the DeLong test.

**Results:**

The nomogram achieved AUCs of 0.995 (95% CI: 0.989–1.000) in training, 0.959 (95% CI: 0.910–1.000) in validation, and 0.894 (95% CI: 0.807–0.981) in test cohorts. Our nomogram significantly outperformed clinical, radiomics, and DTL models, as well as physician assessments (senior physicians: 0.693, *p* = 0.001; junior physicians: 0.600, *p* < 0.001).

**Conclusion:**

This multi-omics nomogram, integrating HIM-derived, clinical, radiomic, and DTL features, accurately predicts post-MT discharge outcomes, enabling early identification of high-risk patients and optimizing management to improve prognosis.

## 1 Introduction

The widespread adoption of mechanical thrombectomy (MT) has significantly improved vessel recanalization rates in patients with acute large vessel occlusive stroke. Nevertheless, despite these advancements, more than 50% of patients continue to experience moderate-to-severe disability, as indicated by a modified Rankin Scale (mRS) score greater than 2 at 90 days postoperatively (Morsi et al., 2024). Obtaining reliable 90-day mRS data from stroke survivors is challenging, requiring substantial medical resources and often leading to delays that may hinder timely rehabilitation. Early identification of high-risk individuals and targeted interventions are essential for optimizing rehabilitation resource allocation and improving long-term outcomes (Li et al., 2024; Taleb et al., 2023). Traditional prognostic models, such as the MT-DRAGON and ASTRAL scores, rely primarily on preoperative clinical and imaging parameters and use linear regression methods, which are insufficient for capturing the complex and dynamic biological changes after MT (Cooray et al., 2016). Moreover, many studies focus on 90-day outcome assessments while overlooking the predictive value of discharge outcomes. Clinical evidence shows a strong correlation between discharge status and 90-day prognosis, with patients who have poor discharge outcomes being highly likely to experience unfavorable long-term outcomes, underscoring the importance of early intervention (Cooray et al., 2016; ElHabr et al., 2021; Karamchandani et al., 2021; Chen et al., 2022). Therefore, there is an urgent need for a predictive tool that can identify patients at risk of poor discharge outcomes, enabling timely interventions to improve long-term prognoses.

Hyperattenuating imaging markers (HIM) have become a focal point in prognostic modeling (Dong et al., 2024). Non-contrast computed tomography (NCCT) performed immediately after mechanical thrombectomy detects HIM in 31 to 84% of cases (Lummel et al., 2014). These hyperdense findings typically indicate blood–brain barrier disruption, most often due to contrast extravasation and, less commonly, post-procedural hemorrhage (Hu et al., 2025). Several studies have identified HIM as a key predictor of increased mortality and poor outcomes following MT, highlighting its value as a prognostic marker (Chen et al., 2019; Jiang et al., 2021). Integrating HIM-based predictive models with artificial intelligence (AI) enhances the prediction of post-thrombectomy complications by analyzing complex imaging patterns and multidimensional data, improving accuracy and sensitivity beyond traditional methods (Hu et al., 2024; Song et al., 2025). This approach provides a more robust framework for clinical decision-making, translating imaging insights into actionable strategies.

Artificial intelligence, including both deep transfer learning (DTL) and radiomics, has emerged as a promising tool for predicting outcomes in stroke patients following MT, offering high precision and the potential to enhance diagnostic and therapeutic strategies. Deep learning facilitates multi-level feature extraction directly from raw imaging data, demonstrating significant predictive capabilities (Liu Y. et al., 2023). Similarly, radiomics has garnered attention for its ability to analyze imaging features and predict functional outcomes post-MT (Heo et al., 2019). While both approaches have robust strengths, their comparative performance depends on the context, with neither consistently outperforming the other (Liu W. et al., 2023). The present study aims to integrate DTL, radiomics, and clinical features, combining the strengths of multiple models to achieve more accurate predictions of discharge outcomes in stroke patients post-MT. This integrated approach seeks to optimize resource allocation, enable early personalized interventions, and improve rehabilitation planning, ultimately enhancing long-term prognosis.

## 2 Methods

### 2.1 Ethical approval of the study protocol

This study was approved by the Ethics Committee (Approval Number: K2024139). During the prospective phase, the model was deployed without any clinical interventions or changes to standard care. After the prospective period, data were retrieved from the research database, along with our model’s predictions, for downstream analysis. All clinical investigations were conducted under the principles outlined in the Declaration of Helsinki.

### 2.2 Patient selection and study design

**Figure 1:**
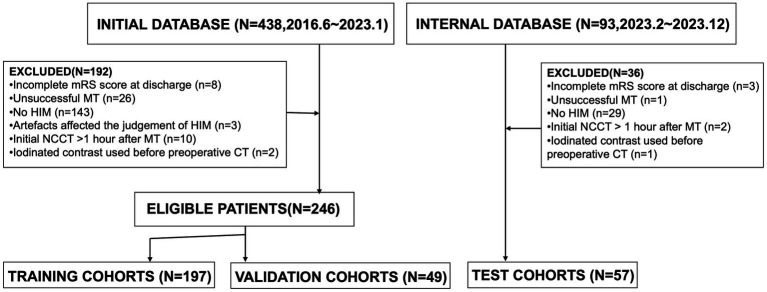
The patient selection flowchart.

We retrospectively reviewed patients diagnosed with acute ischemic stroke due to intracranial large vessel occlusion (LVO) who underwent endovascular MT between June 2016 and December 2023. The indications and contraindications for MT and thrombolysis were based on the most current guidelines available at the time of treatment. General clinical characteristics, laboratory findings, clinical presentations, and imaging data were collected. Inclusion criteria were as follows: (1) patients underwent head NCCT post-MT; (2) initial postoperative NCCT was performed within 1 h after MT; (3) HIM, defined as hyperattenuation in the brain parenchyma or subarachnoid space, were detected on the initial post-MT NCCT. Exclusion criteria included: (1) incomplete mRS score at discharge; (2) unsuccessful MT, such as guidewire failure to reach the occlusion site or angiography confirming vessel recanalization; (3) absence of HIM; (4) imaging artifacts compromising HIM assessment; (5) administration of iodinated contrast before preoperative CT. A flowchart of the study population is presented in Figure 1.

### 2.3 Imaging

Initial postoperative non-enhanced head CT scans were acquired within 1 h post-thrombectomy using either a 64-row spiral CT scanner (Somatom® Definition AS, Siemens Healthineers, Forchheim, Germany) or a 62-row spiral scanner (Optima® CT620, GE Medical Systems, Milwaukee, WI, USA). Scanning parameters were as follows: axial mode, tube voltage of 120 kV, tube current of 250–300 mAs, coverage from the skull base to the cranial vertex, a section thickness of 5 mm, and reconstruction with a standard algorithm. Two neuroradiologists with over 10 years of experience and blinded to the clinical data independently evaluated imaging data in randomized order. Any discrepancies were resolved through consensus discussion.

**Figure 2:**
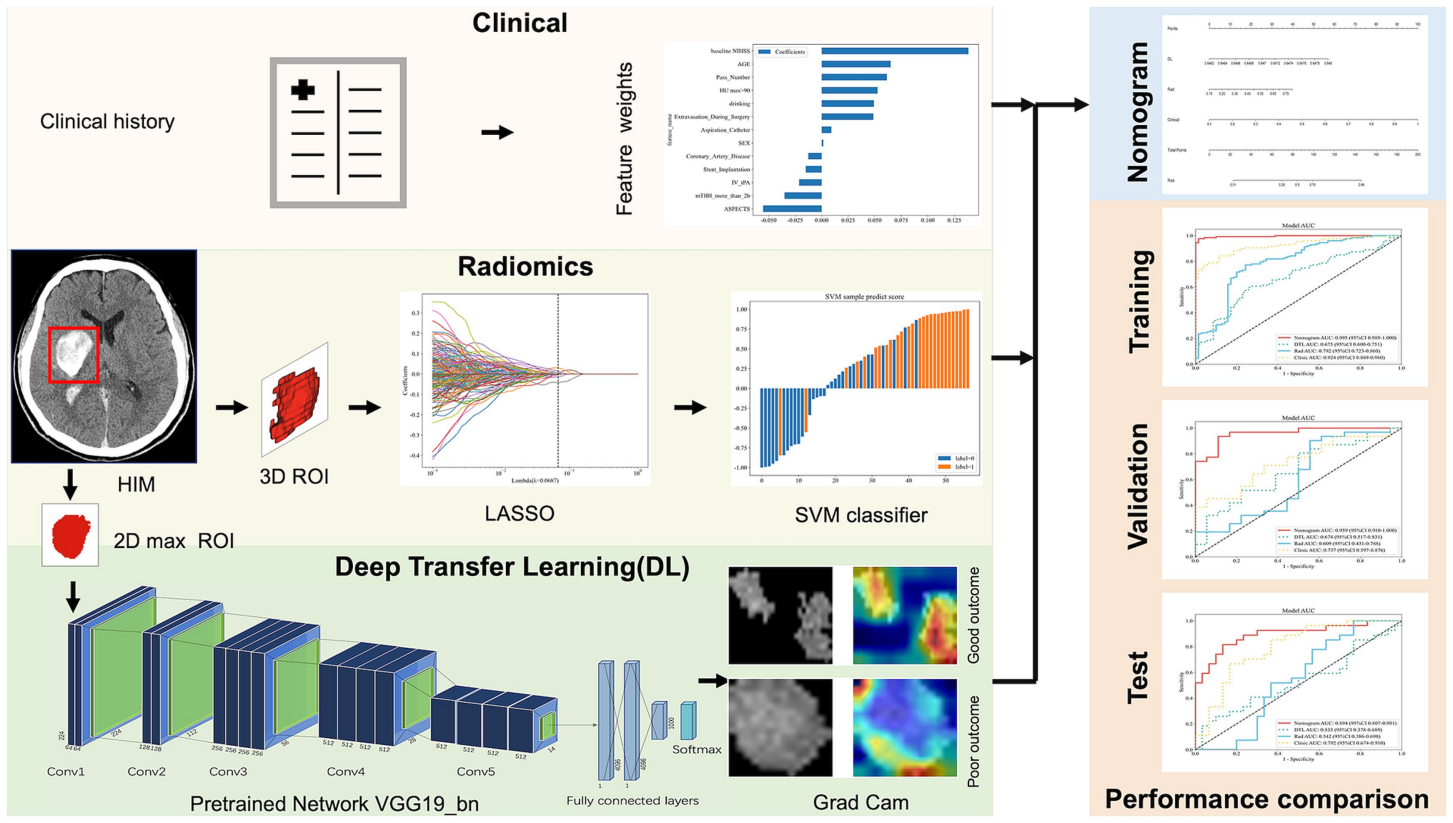
The overall framework of the proposed model. ROI, region of interest; LASSO, least absolute shrinkage and selection operator; SVM, support vector machine; Grad Cam, Gradient-weighted Class Activation Mapping; AUC, area under curve.

The workflow and global analysis pipeline for the classification model are illustrated in Figure 2. HIM was manually delineated on each CT image, and the region of interest (ROI) encompassing the HIM was extracted for input into the predictive model. To ensure the reliability and reproducibility of radiomics features, 30 lesions were randomly selected, and intra-class correlation coefficients (ICC) were used to assess inter- and intra-observer agreement in feature extraction. Reader A performed ROI segmentation twice, with a 1-month interval, to evaluate intra-observer agreement. Reader B segmented the 30 lesions once, and the extracted radiomics features were used to assess inter-observer agreement. Features with ICC > 0.75 were deemed reliable and retained for model construction, resulting in 1,725 features with excellent agreement (ICC > 0.75). Additionally, segmentation consistency was quantified using the Dice similarity coefficient (DSC), yielding a mean DSC of 0.954, indicating high agreement between the two neuroradiologists. DTL and radiomics features were extracted separately: DTL features were derived from the largest cross-sectional ROI, while radiomics features were computed from the entire ROI volume. Feature selection was conducted sequentially using analysis of variance (ANOVA), Pearson’s correlation coefficient, and the least absolute shrinkage and selection operator (LASSO).

### 2.4 Data collection

In this study, 303 patients were enrolled from June 2016 to December 2023. Of these, 246 patients treated between June 2016 and January 2023 were allocated to model training and validation: 197 (80%) were randomly assigned to the training cohort, and 49 (20%) to the validation cohort. An additional 57 patients from February 2023 to December 2023 were included in the internal test cohort. Functional outcomes at discharge were evaluated using the mRS (range: 0–6), with unfavorable outcomes defined as mRS scores of 4–6, extracted from medical records by trained clinicians. Discrepancies were resolved through consultation.

By reviewing patient charts and procedure notes, we gathered the following clinical indicators and surgical details: age, sex, hyperlipidemia, hypertension, diabetes mellitus, coronary artery disease, atrial fibrillation, history of prior stroke, history of alcohol consumption or smoking, baseline National Institutes of Health Stroke Scale (NIHSS) scores, length of hospital stay, pre-stroke mRS scores, admission mRS scores, thrombolysis status, stent implantation, modified Thrombolysis in Cerebral Infarction (mTICI) scores, use of aspiration catheters, and number of thrombectomy attempts. Imaging metrics, including the Alberta Stroke Program Early CT Score (ASPECTS) for anterior and posterior circulation, subarachnoid HIM, HUmax values, and other relevant parameters, were determined through consensus between two neuroradiologists, each with over 5 years of experience and blinded to the patients’ clinical information.

### 2.5 Feature extraction and selection

Radiomic features were extracted from HIM on NCCT using an in-house feature analysis program based on Pyradiomics^1^. To eliminate any potential variations in CT images obtained using different CT scanners, NCCT images were reconstructed using a voxel size of 1 × 1 × 1 mm^3^ and gray-scale discretization. A total of 1,834 radiomic features were computed, covering shape, first-order statistics, gray-level co-occurrence matrix, gray-level run-length matrix, gray-level size zone matrix, neighboring gray-tone difference matrix, gray-level dependence matrix, and wavelet features.

DTL features were extracted using five models: VGG19_bn, Inception v3, ResNet50, VGG16, and ResNet152. Among these, we selected the model with the highest predictive performance for this study, based on its superior area under the curve (AUC) value. Pre-trained on large-scale datasets, the selected model was fine-tuned using the maximum cross-sectional HIM ROI. Data augmentation techniques, including RandomResizedCrop and RandomHorizontalFlip, were applied to improve generalization. Features from the ‘avgpool’ layer generated 25,088 DTL features, which were then reduced to 303 using principal component analysis (PCA). Radiomics features were extracted and evaluated using five machine learning models: SVM, KNN, Random Forest, XGBoost, and LightGBM. Among these, SVM performed best, achieving an AUC of 0.609. As a result, a clinical-radiomics-DTL nomogram was constructed using SVM. Clinical, radiomics, and DTL features were standardized to have a mean of 0 and a variance of 1. Feature selection was performed using ANOVA, Pearson’s correlation coefficient, PCA, and LASSO to enhance the models’ generalization ability and reduce overfitting. For the clinical, radiomics, and DTL models, ANOVA, Pearson’s correlation coefficient, and LASSO were applied sequentially to complete feature selection. PCA, ANOVA, Pearson’s correlation coefficient, and LASSO were used sequentially for the combined model.

### 2.6 Development of the clinical signature

To develop the clinical model, we first performed univariate logistic regression analysis to identify clinical variables significantly associated with the outcome. Variables with a *p*-value less than 0.05 were considered candidate predictors. These selected features were then used to construct the clinical model using a support vector machine (SVM) algorithm, implemented in Python (version 3.11.13). Model performance was evaluated in the training and test cohorts using receiver operating characteristic (ROC) curves and corresponding AUC values.

### 2.7 Model development and validation

A nomogram integrating clinical, radiomics, and DTL features was developed, alongside separate clinical, radiomics, and DTL models for comparison. Model performance was assessed using sensitivity, specificity, receiver operating characteristic (ROC) curves, and AUC. Five-fold cross-validation, combined with grid search, was employed to optimize the hyperparameters of the SVM model. The training cohort data were divided into five folds, yielding five prediction models. For each model, sensitivity, specificity, and AUC were computed. The fold with the median AUC was selected as the representative split. Feature engineering was strictly limited to the training data in each fold to prevent data leakage and ensure unbiased evaluation. Models were trained and evaluated on the training, validation, and test cohorts. The Shapley Additive Explanations (SHAP) method was used to visualize and quantify each feature’s contribution to model predictions. Calibration curves were generated to assess the agreement between predicted probabilities and observed outcomes in the test cohort. The DeLong test was applied to compare ROC curve performance across models, and decision curve analysis (DCA) was performed to evaluate the clinical utility of the predictive models.

### 2.8 Implementation and hardware

The network architecture was implemented using Python and the PyTorch library. The DTL model was trained with the stochastic gradient descent algorithm as the optimizer, using a learning rate of 0.001, a mini-batch size of 8, and a binary cross-entropy loss function. Batch normalization was applied after each convolution layer to accelerate convergence and reduce overfitting. The network was trained for 50 epochs.

### 2.9 Sensitivity analysis of nomogram performance using 90-day mRS outcomes

To assess the consistency and robustness of the nomogram in predicting longer-term outcomes, a sensitivity analysis was conducted in the test cohort using 90-day mRS scores as the outcome measure. Patients with 90-day mRS scores of 3–6 were classified as having unfavorable outcomes.

### 2.10 Statistical analysis and performance evaluation

Statistical analyses were performed using SPSS software (version 27.0, IBM). Continuous variables are reported as means with standard deviations, and categorical variables as frequencies and percentages. The Kruskal–Wallis test was used to compare age, hospital stay duration, baseline NIHSS score, discharge NIHSS score, admission mRS score, and ASPECTS. Other categorical variables were analyzed using the chi-square test or Fisher’s exact test, as appropriate. Feature extraction, selection, and model construction were conducted using Python (version 3.11.13). Model performance was evaluated by calculating the AUC. Calibration curves were generated to assess agreement between predicted probabilities and observed outcomes in the test cohort. The DeLong test compared performance differences between ROC curves of different models, and DCA evaluated the clinical utility of the prediction models. A *p*-value < 0.05 was considered statistically significant for all tests.

## 3 Results

### 3.1 Cohort and clinical characteristics

**Figure 3:**
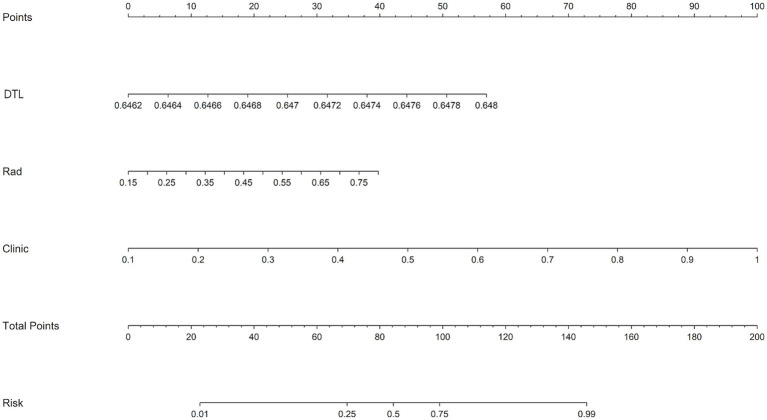
A nomogram integrating radiomic features, deep learning algorithms, and clinical parameters was developed to predict the risk of unfavorable discharge outcomes.

The study included a training cohort of 197 patients, a validation cohort of 49 patients, and a test cohort of 57 patients. Clinical feature selection was performed using ANOVA, Pearson correlation, and LASSO. Table 1 details the patient characteristics. Unfavorable discharge outcomes, defined as mRS 4–6, were observed in 185 of 303 patients. Significant differences among cohorts were identified in hyperlipidemia (*p* = 0.010), coronary artery disease (*p* = 0.040), thrombolysis (*p* = 0.005), stent type, (*p* = 0.001), and aspiration catheter use (*p* < 0.001). A nomogram incorporating clinical, radiomics, and DTL features was developed (Figure 3). To derive the DTL features, we evaluated five convolutional neural network models: VGG19_bn, Inception v3, ResNet50, VGG16, and ResNet152. Among these, VGG19_bn demonstrated the highest performance (AUC = 0.674) and was selected for this study.

**Table 1:** Characteristics of patients in the train, validation and test cohort.

Feature name	Training *n* = 197	Validation *n* = 49	Test *n* = 57	*p*-value
Age (year), mean±SD	66.64 ± 14.58	69.27 ± 12.80	65.84 ± 16.30	0.448
Men, *n* (%)	129 (65.48)	29 (59.18)	36 (63.15)	0.705
Atrial fibrillation, *n* (%)	80 (40.60)	18 (36.73)	24 (42.10)	0.842
Hypertension, *n* (%)	110 (55.83)	36 (73.46)	35 (61.40)	0.076
Hyperlipidemia, *n* (%)	14 (7.10)	6 (12.24)	12 (21.05)	0.010^*^
Diabetes Mellitus, *n* (%)	33 (16.75)	10 (20.40)	9 (15.78)	0.794
Coronary artery disease, *n* (%)	22 (11.16)	11 (22.44)	4 (7.01)	0.040^*^
Drinking, *n* (%)	43 (21.82)	11 (22.44)	10 (17.54)	0.760
Smoking, *n* (%)	43 (21.82)	13 (26.53)	12 (21.05)	0.750
Thrombolysis, *n* (%)	76 (38.57)	19 (38.77)	9 (15.78)	0.005^*^
Pre-stroke mRS ≤ 2, *n* (%)	194 (98.47)	49 (100.00)	57 (100.00)	0.444
Prior stroke, *n* (%)	28 (14.21)	8 (16.32)	9 (15.78)	0.911
Baseline NIHSS, median (Q1, Q3)	16 (12–21)	15 (12–20)	16 (10–20)	0.750
ASPECTS, median (Q1, Q3)	9 (8–10)	9 (8–10)	9 (8–10)	0.422
HUmax≥90, *n* (%)	56 (28.42)	15 (30.61)	18 (31.57)	0.880
Anterior Circulation, *n* (%)	184 (93.40)	44 (89.79)	54 (94.73)	0.579
sHIM, *n* (%)	76 (38.57)	17 (34.69)	20 (35.08)	0.819
mTICI more than 2b, *n* (%)	176 (89.34)	44 (89.79)	53 (92.98)	0.718
Stent Type, *n* (%)				0.001^*^
Solitaire	116 (58.88)	24 (48.97)	36 (63.15)	
Trevo	30 (15.22)	12 (24.48)	0 (0.00)	
Solitaire+Trevo	34 (17.25)	5 (10.20)	19 (33.33)	
Others	16 (8.12)	8 (16.32)	2 (3.50)	
Pass number, median (Q1, Q3)	1 (1–2)	2 (1–3)	1 (0–2)	0.313
Aspiration catheter, *n* (%)	63 (31.97)	20 (40.81)	47 (82.45)	0.000^*^
Stent implantation, *n* (%)	38 (19.28)	11 (22.44)	7 (12.28)	0.358
LOS, median (Q1, Q3)	10 (7–19)	12 (7–16)	11 (7–16)	0.985
HT, *n* (%)	136 (69.03)	38 (77.55)	36 (63.15)	0.275
mRS at admission, *n* (%)				0.567
0	1 (0.51)	0 (0.00)	0 (0.00)	
1	1 (0.51)	0 (0.00)	1 (1.75)	
2	5 (2.54)	0 (0.00)	0 (0.00)	
3	12 (6.09)	4 (8.16)	4 (7.02)	
4	36 (18.27)	6 (12.24)	11 (19.30)	
5	142 (72.08)	39 (79.59)	41 (71.93)	

SD, standard deviation; NIHSS, National Institutes of Health Stroke Scale; ASPECTS, Alberta Stroke Program Early Computed Tomography Score; Q1, first quartile; Q3, third quartile; HU, Hounsfield; sHIM, subarachnoid hyperattenuated imaging markers; mTICI, modified thrombolysis in cerebral infarction; LOS, length of stay; mRS, modified Rankin Scale; HT, hemorrhagic transformation. *represents *p* < 0.05.

### 3.2 Performance of models in the training cohort

**Figure 4:**
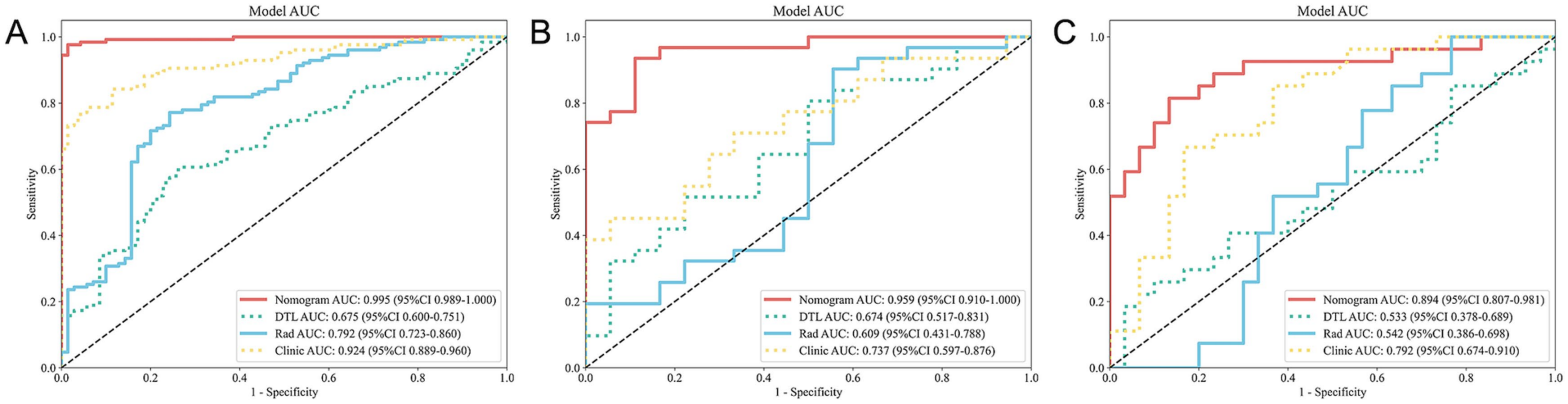
ROC curves for all models across the training **(A)**, validation **(B)**, and test **(C)** cohorts. ROC, receiver operating characteristic.

In the training cohort, the nomogram demonstrated superior performance with an AUC of 0.995 (95% CI: 0.989–1.000), a sensitivity of 0.969, and a specificity of 0.986. The clinical model achieved an AUC of 0.924 (95% CI: 0.889–0.960), while the radiomics model and DTL model yielded AUCs of 0.792 (95% CI: 0.723–0.860) and 0.675 (95% CI: 0.600–0.751), respectively (Figure 4A).

### 3.3 Performance of models in the validation cohort

In the validation cohort, the nomogram maintained high performance with an AUC of 0.959 (95% CI: 0.910–1.000), a sensitivity of 0.903, and a specificity of 0.889. The clinical model achieved an AUC of 0.737 (95% CI: 0.597–0.876), while the radiomics model and DTL model yielded AUCs of 0.609 (95% CI: 0.431–0.788) and 0.674 (95% CI: 0.517–0.831), respectively (Figure 4B).

### 3.4 Performance of 5-fold cross-validation

Five independent validation results were obtained, as shown in Table 2. The fold with the median AUC (0.959) was selected to represent a typical data split for model training and evaluation.

**Table 2:** Performance metrics from 5-fold cross-validation.

Fold	AUC	95% CI	Sensitivity	Specificity
1	0.943	0.8835–1.0000	0.833	0.950
2	0.956	0.9064–1.0000	0.806	1.000
3	0.959	0.9096–1.0000	0.903	0.889
4	0.966	0.9193–1.0000	0.848	1.000
5	0.972	0.9351–1.0000	0.833	1.000

AUC, Area Under the Curve; CI, Confidence Interval.

### 3.5 Performance of models in the test cohort

**Figure 5:**
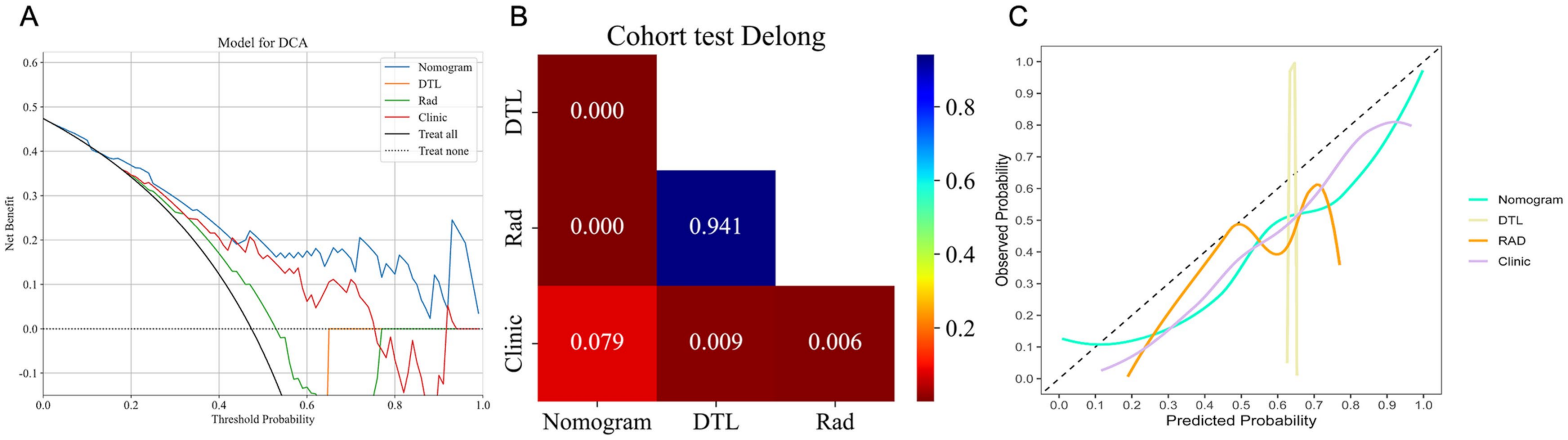
The DCA curves **(A)**, DeLong test results **(B)**, and calibration curves **(C)** for all models in the test cohort. **(A)** DCA curves of different models in the test cohort. The y-axis represents the net benefit, and the x-axis represents the threshold probability. DCA, Decision Curve Analysis. **(B)** A significant difference between models was determined by the DeLong test (*p* < 0.05). **(C)**. Calibration curves for models in the test cohort. The x-axis represents predicted probabilities of unfavorable outcomes, and the y-axis represents observed probabilities.

In the test cohort, our nomogram achieved an AUC of 0.894 (95% CI: 0.807–0.981), outperforming the clinical model (AUC 0.792, 95% CI: 0.674–0.910), the radiomics model (AUC 0.542, 95% CI: 0.386–0.698), and the DTL model (AUC 0.533, 95% CI: 0.378–0.689) (Figure 4C). DCA further revealed that all models improved outcome predictions compared to no-model scenarios, with the nomogram providing the most significant clinical benefit (Figure 5A). To evaluate the models’ effectiveness, the DeLong test was performed (Figure 5B). The findings indicated that the AUC of the nomogram was markedly superior to that of the radiomics model (*p* < 0.001) and the DTL model (p < 0.001) within the testing cohort. Moreover, the nomogram displayed a trend toward enhanced performance relative to the clinical model (*p* = 0.079), though this difference fell short of statistical significance. To assess the agreement between predicted and observed outcomes for the nomogram, calibration curves were generated (Figure 5C).

### 3.6 Enhancing model interpretability using SHAP values

**Figure 6:**
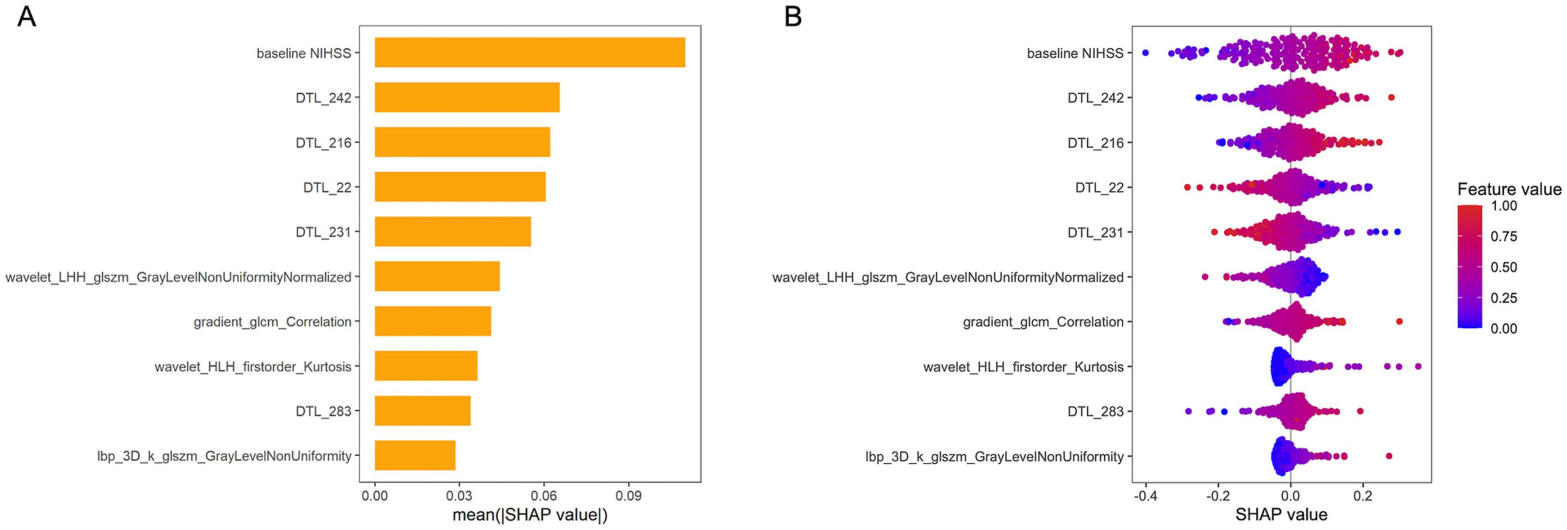
SHAP values for interpreting the combined model. **(A)** Bar plot illustrating the relative contributions of features to the model’s output. The top-ranked feature, “Baseline NIHSS” exhibits the greatest predictive power, significantly outperforming lower-ranked features. **(B)** Beeswarm plot depicting SHAP values for model features. Positive SHAP values indicate a higher predicted risk of unfavorable outcome, while negative values correspond to a lower risk. NIHSS, National Institutes of Health Stroke Scale; DTL, deep transfer learning; LHH, low-high-high-pass filtered image; GLSZM, gray level size zone matrix; GLCM, gray-level co-occurrence matrix; HLH, high-low–high filtered image.

Figure 6 presents a SHAP bar plot, ranking features by their contribution to the model’s output in descending order, and a SHAP beeswarm plot, illustrating the impact and distribution of each feature on model predictions. The magnitude of SHAP values indicates the strength of each feature’s influence on individual predictions.

### 3.7 Sensitivity analysis using 90-day mRS outcomes

We performed a sensitivity analysis in the test cohort using 90-day mRS scores as the outcome measure, despite one patient being lost to follow-up at 90 days. The nomogram achieved an AUC of 0.854, suggesting good discriminatory ability for predicting long-term functional disability.

### 3.8 Clinically actionable threshold and risk stratification

To enhance the nomogram’s clinical utility, a probability threshold of 0.760 was established by maximizing the Youden Index (0.645) in the test cohort. At this cutoff, the model achieved a sensitivity of 0.778, specificity of 0.867, positive predictive value (PPV) of 0.840, and negative predictive value (NPV) of 0.812, striking an optimal balance between false positives and false negatives. The positive likelihood ratio (PLR) of 5.85 and negative likelihood ratio (NLR) of 0.26 demonstrate robust risk stratification.

### 3.9 Performance comparison of nomogram and physicians

**Figure 7:**
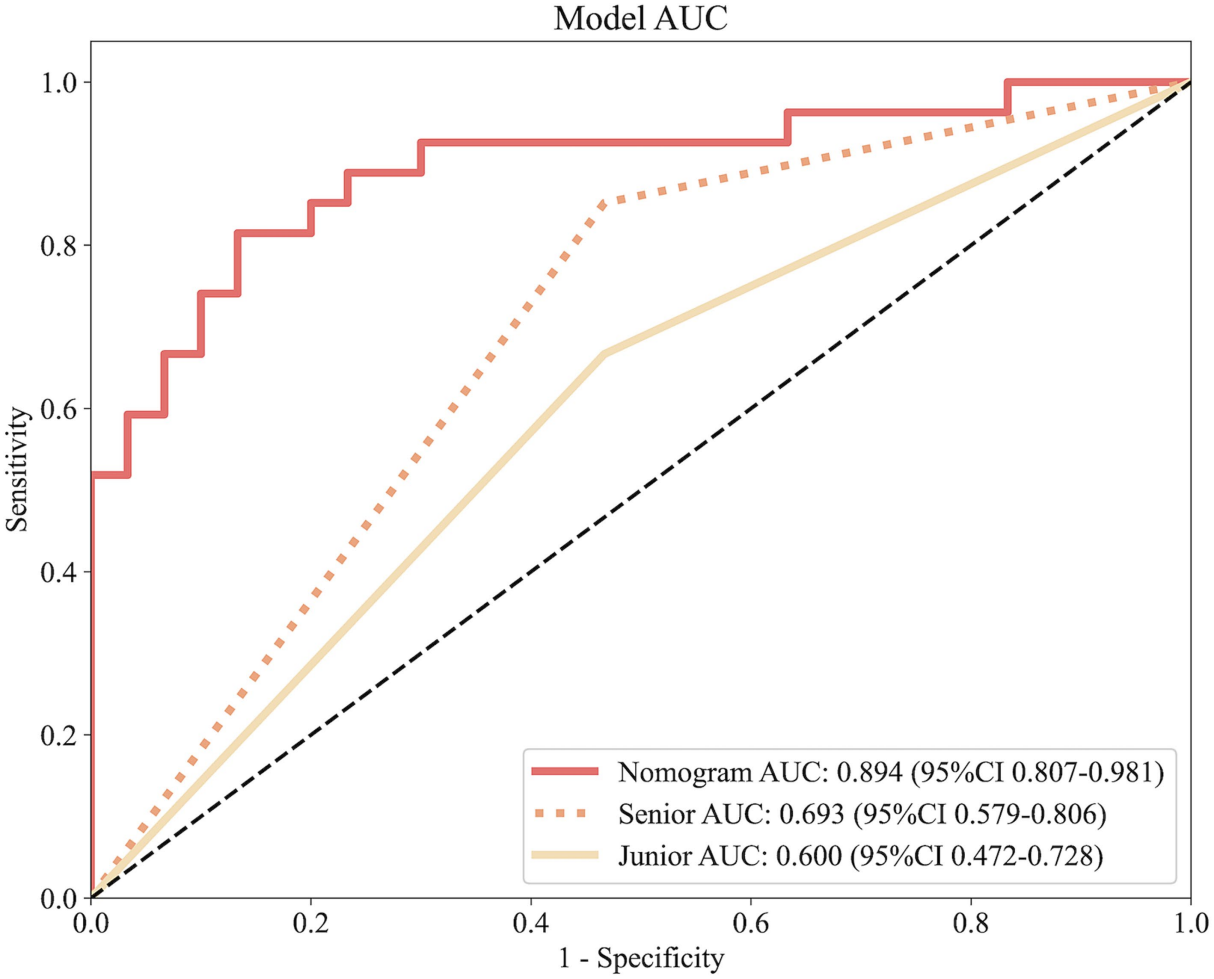
ROC curves comparing predictive performance among the nomogram, a senior physician, and a junior physician.

Figure 7 illustrates the ROC curves for the nomogram and physicians. The nomogram achieves an AUC of 0.894, outperforming both the senior physician (0.693) and the junior physician (0.600). In comparison to the senior physician, the nomogram demonstrates superior accuracy (83% vs. 53%), specificity (83% vs. 80%), and sensitivity (82% vs. 62%). Similarly, when compared to the junior physician, the nomogram exhibits greater accuracy (83% vs. 53%), specificity (83% vs. 64%), and sensitivity (82% vs. 56%).

## 4 Discussion

Herein, we extracted radiomics and DTL features from HIM on post-thrombectomy NCCT, integrated them with clinical data, and developed a nomogram to predict discharge prognosis. Importantly, our nomogram achieved an AUC of 0.894 in a prospective test cohort, surpassing single-modality models and demonstrating its superior predictive performance for discharge outcomes.

We defined an unfavorable discharge outcome as an mRS score of 4–6. Several studies have previously employed the mRS at discharge as a prognostic tool for assessing early outcomes following ischemic or hemorrhagic stroke treatment, with some establishing an mRS score greater than 3 as the threshold for an unfavorable prognosis (Hallevi et al., 2009; Shi et al., 2018; Zhao et al., 2023). Among the clinical variables in our nomogram, the baseline NIHSS score was the most influential predictor of discharge prognosis following MT, consistent with prior findings (Zhang et al., 2025). Baseline NIHSS scores, which measure stroke severity, reliably predict outcomes in large vessel occlusion, with higher scores indicating greater neurological deficits and worse prognosis (Li et al., 2018). Although the nomogram demonstrated stronger predictive performance than the clinical model in the test cohort, the difference in AUC between the two was not statistically significant (*p* = 0.079). The relatively strong performance of the clinical model alone may be attributed to its inclusion of the most influential predictor—baseline NIHSS—which captures a substantial amount of prognostic information and thereby enhances its predictive accuracy.

Our model not only incorporates clinical indicators but also integrates deep learning and radiomics models to form a robust predictive framework. Deep learning models have demonstrated strong performance in prognostic prediction. Nishi and Dipros previously utilized the convolutional neural network (CNN) and 3D DenseNet121 deep learning models to predict functional outcomes 3 months after MT, achieving an AUC of 0.81 (Nishi et al., 2020; Diprose et al., 2025). In contrast, our study leveraged HIM features extracted from post-thrombectomy CT scans. These CT images, acquired 1 hour after the procedure, effectively capture reperfusion injury while minimizing the confounding influence of preoperative variability (Bai and Lyden, 2015). Moreover, our model demonstrates superior predictive performance compared to previous approaches. Here, we employed the VGG19_BN model, leveraging its stacked 3 × 3 convolutional kernels to deepen the network and enhance feature extraction capabilities. The batch normalization in VGG19_BN effectively reduces internal covariate shift, accelerates training, and stabilizes gradient flow, thereby improving prognostic accuracy (Han et al., 2024). Radiomics, widely applied in predicting stroke discharge outcomes, provides quantitative insights through feature extraction and has shown considerable promise in forecasting prognosis after thrombectomy. Evidence suggests that models integrating deep learning and radiomics significantly outperform standalone deep learning approaches due to their superior image feature extraction capabilities (Liu W. et al., 2023). Thus, our nomogram, which unifies clinical, deep learning, and radiomics features, establishes a robust predictive framework that enhances accuracy and supports personalized treatment strategies.

We acknowledge that the discharge mRS, while valuable for early prognostication, may not fully capture long-term functional outcomes. To explore the relationship between discharge status and 90-day prognosis, we conducted a sensitivity analysis using 90-day mRS scores as the endpoint in the test cohort. Despite the shift to a longer-term outcome measure, the model maintained robust discriminative ability (AUC = 0.854), confirming its stability and generalizability. This finding suggests a potential link between an unfavorable discharge outcome (mRS > 3) and an unfavorable 90-day prognosis (mRS > 2), indicating that clinicians could leverage this early marker to identify high-risk patients and implement targeted interventions to improve long-term outcomes.

**Figure 8:**
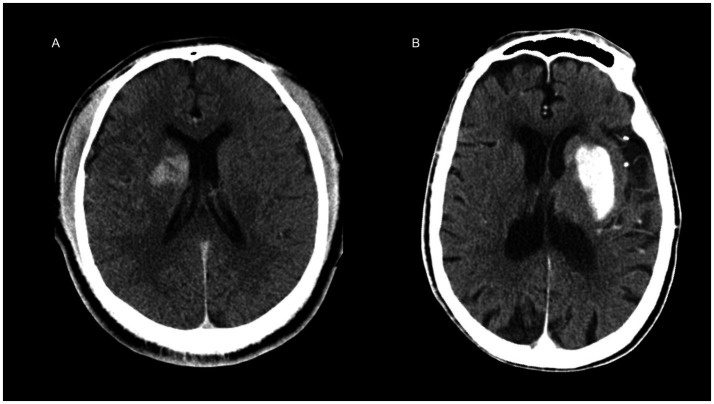
Representative examples illustrating the clinical application of the nomogram. **(A)** A 31-year-old male patient with a baseline NIHSS score of 11. The non-contrast CT image acquired immediately after thrombectomy is shown. The nomogram-predicted risk score was 0.444 (below the clinical threshold of 0.760), indicating a favorable discharge outcome, which was consistent with the actual prognosis. **(B)** A 93-year-old male with a baseline NIHSS score of 21. The immediate post-thrombectomy non-contrast CT is presented. The nomogram predicted a risk score of 0.964, exceeding the 0.760 threshold, suggesting a high risk of poor discharge prognosis, which aligned with the clinical outcome.

To demonstrate the clinical utility of the nomogram, we identified a probability threshold of 0.760 that effectively stratifies patients by their risk of poor functional outcome at discharge (Figure 8). Patients with predicted probabilities above 0.760 warrant intensified neurological monitoring and early, personalized rehabilitation to mitigate secondary decline and improve recovery, while those below this threshold may receive standard post-thrombectomy care, optimizing resource allocation.

We evaluated the predictive performance of our nomogram for discharge outcomes against that of senior and junior physicians. Using their extensive experience and deep familiarity with critical indicators, senior physicians surpassed junior physicians in performance but faced challenges in handling high-dimensional data or discerning subtle patterns, areas in which the model excelled. This highlights the potential of integrating machine learning models with clinical expertise to enhance prognostic accuracy and decision-making (Rajpurkar et al., 2017). This multi-omics model effectively captures complex biological and imaging patterns by incorporating DTL-derived imaging features, traditional radiomics, and clinical variables, offering objectivity and consistency that surpasses human capability (Choi et al., 2023). The nomogram could serve as a reliable decision-support tool in the future, enhancing clinical precision by integrating real-time patient data with physician expertise, particularly benefiting junior physicians and time-sensitive scenarios.

Nonetheless, the present study has several limitations. Firstly, it was conducted at a single center using two scanners with standardized protocols, which minimizes intra-institutional variability but may limit generalizability to diverse clinical settings. Variations in scanner vendors, acquisition protocols, and patient populations across institutions could impact feature robustness and model performance. To address this, we plan prospective multicenter studies with diverse imaging sources and populations to evaluate model transferability and clinical utility in future. Secondly, the small sample size may reduce statistical power and external validity, particularly in the prospective test cohort. Thirdly, while discharge outcomes are closely related to 90-day prognosis, this association is not absolute and requires further validation. Finally, while manual lesion segmentation by experienced neuroradiologists ensures rigor, it introduces subjectivity and variability, particularly for poorly defined lesions, potentially biasing our results. To address this, future research should explore automated segmentation techniques to enhance reproducibility and reduce variability. Therefore, future multicenter studies with larger, more diverse cohorts and automated feature extraction are warranted to address these limitations and further validate our model’s clinical utility.

## 5 Conclusion

In conclusion, our study introduces a robust predictive model that effectively integrates radiomics, deep learning, and clinical data to predict patient outcomes following thrombectomy. The high predictive accuracy of our nomogram makes it a promising clinical decision-making tool, with the potential to enhance patient prognosis and improve personalized treatment strategies.

## Data Availability

The raw data supporting the conclusions of this article will be made available by the authors, without undue reservation.

## Footnotes

^1^ http://pyradiomics.readthedocs.io
